# The role of self-stigma in mediating the association between externalizing and treatment-seeking intention

**DOI:** 10.3389/fpsyg.2025.1686583

**Published:** 2025-12-12

**Authors:** Brianna N. Davis, Laura E. Drislane

**Affiliations:** Department of Psychology, University of Mississippi, Oxford, MS, United States

**Keywords:** self-stigma, externalizing proneness, treatment-seeking intention, structural equation modeling, mediation

## Abstract

**Introduction:**

Self-stigma occurs when an individual internalizes and accepts the stereotypes and negative views of mental illness endorsed by the public. High levels of self-stigma negatively predict treatment-seeking intention, but little work has examined how self-stigma functions specifically among individuals with tendencies toward impulsivity.

**Methods:**

The current study implemented structural equation modeling (SEM) to investigate whether self-stigma as operationalized by the MHSIS model mediated the relationship between externalizing proneness and the intention to seek mental health treatment. Data were collected from community participants (*N* = 394) preselected for the presence of impulse control problems (e.g., ADHD, alcohol use) and mental health concerns.

**Results:**

Externalizing was weakly negatively associated with the intention to seek treatment. Externalizing proneness was associated with higher levels of self-stigma, particularly at the later stages (i.e., application); however, self-stigma did not appear to mediate the association between externalizing and reduced treatment seeking intention.

**Discussion:**

This study informs how self-stigma exists across the externalizing spectrum and may serve as a transdiagnostic target for intervention.

## Introduction

Mental illness is a public health crisis. Around 50% of adults living in the United States have experienced a mental illness in their lifetime (Kessler et al., 2005), and approximately 21% have a diagnosed mental illness in any given year (Center for Behavioral Health Statistics and Quality, 2016). Mental illness is associated with serious deleterious functional outcomes such as higher rates of suicide (Bachmann, 2018), incarceration (Bronson et al., 2017), heart disease (Rossom et al., 2022), and unemployment (Center for Behavioral Health Statistics and Quality, 2016). Engagement in empirically supported treatments is an important component of care for reducing the severity of mental illnesses (van Dis et al., 2020) and alleviating the associated individual, community, and societal impacts.

However, the majority of people with mental illness do not receive treatment (Thornicroft, 2007), due to a complex combination of individual, societal, or environmental barriers such as low perceived need, lack of available care, or cost (Mojtabai et al., 2011; Sareen et al., 2007). Those who ultimately do receive care show on average an 11-year span between the onset of symptoms and treatment (Wang et al., 2004). Reducing mental health disparities will require sweeping structural-level changes; however, it is also crucial to identify modifiable individual risk factors that contribute to barriers in seeking treatment, which may be addressed on a more immediate, individual-level basis. One potentially relevant factor is self-stigma, which is defined as the extent to which an individual internalizes negative public stereotypes or attitudes about stigmatized identities, including mental illness (Corrigan and Rao, 2012). Previous studies have examined self-stigma in relation to specific disorder presentations; however, no studies have attempted to assess how self-stigma relates to broader psychopathology spectra such as the externalizing spectrum. Self-stigma may serve as a mediator between broad propensities toward mental illness and the intention to seek treatment and thus could be targeted to increase treatment utilization.

The public holds a range of negative stereotypes about individuals living with mental illness (e.g., they are dangerous or incompetent). This “public stigma” can be distinguished from the phenomenon of “self-stigma” which occurs among people with mental illness who internalize and begin to endorse these same harmful beliefs (Corrigan and Watson, 2002). Self-stigma decreases self-esteem and self-efficacy and is a known barrier to treatment-seeking intention (Corrigan et al., 2009a; Lannin et al., 2016; Kao et al., 2016). Higher levels of self-stigma predict worsening mental health symptoms and lower morale (Ritsher and Phelan, 2004), poorer medication adherence (Uhlmann et al., 2014), and reduced treatment effectiveness (Ociskova et al., 2015) among individuals diagnosed with a mental illness.

Early conceptualizations equated self-stigma with perceived stigma, one’s expectations of being treated negatively by others due to their identity, such as living with a mental illness (i.e., one’s awareness of public stigma; Link, 1987). However, perceived stigma does not assess the extent to which stigmatized individuals accept and endorse these beliefs themselves, nor does it explain the process by which self-stigma leads to negative consequences, such as decreased self-esteem and self-efficacy. Thus, Corrigan and Rao (2012) introduced the stage model of self-stigma to more clearly delineate the steps by which public stigma is internalized. According to this model, self-stigma is a construct that progresses in stages delineated by an individual’s internal awareness, acceptance, application, and harm to self of the stereotypes or attitudes endorsed by the public (Corrigan and Rao, 2012). The model assumes that merely being aware of stigma is not sufficient to negatively impact a person’s self-esteem or self-efficacy; it is only when an individual begins to apply these negative beliefs about mental illness to themselves that the deleterious effects will be observed.

The first stage of self-stigma, awareness, occurs when an individual is cognizant that stigma exists (e.g., “I have heard that people with mental illness are crazy;” Corrigan and Rao, 2012). The second stage, agreement, materializes when an individual accepts that stigma as truth (e.g., “People with mental illness are crazy”). After acceptance of a stigma, an individual may progress toward applying that stigma to themselves (e.g., “I have a mental illness therefore, I am crazy”). The final stage of self-stigma occurs when an individual with mental illness experiences harm to self because of the stigma, which is evidenced by significant decreases in self-esteem and self-efficacy (e.g., “Because I am crazy, I am not good enough”). Consequently, at this final stage of self-stigma, individuals show decreased motivation to progress in their life goals (Corrigan and Rao, 2012). Thus, the greatest harm occurs in the later stages of self-stigma (i.e., the application and “why try” stages). Notably, self-stigma is borne from societal level stereotypes. Frequent or intense exposure to such beliefs can ultimately lead an individual to internalize such beliefs and alter their own thought patterns. From this perspective, the tendency for a person with mental illness to express beliefs which devalue individuals with a mental health challenges could be conceptualized as a maladaptive cognitive style that progresses over time. As such, it is reasonable to assume self-stigma may be modifiable and amenable to cognitive remediation strategies in much the same way as other forms of cognitive distortions (Brazão et al., 2015) such that decreasing self-stigma may therefore improve someone’s willingness to engage in treatment.

Overall, there is strong evidence that self-stigma is present across various mental illness diagnoses (Livingston and Boyd, 2010) and is associated with reduced treatment-seeking among individuals with specific externalizing problems (Ahern et al., 2007; Etesam et al., 2014; Grambal et al., 2016; Rüsch et al., 2006). For example, self-stigma may impact one’s ability to abstain from or decrease use of drugs or alcohol (Brown et al., 2015) and has been identified as a barrier to treatment-seeking among problematic gamblers (Hing et al., 2016). However, another study found that increased rates of self-stigma predicted *higher* rates of the intention to seek treatment among gamblers (Horch and Hodgins, 2015). Young adults with ADHD report higher levels of self-stigma, which in qualitative research has been attributed to peer stigmatization and feeling different or “other” (McKeague et al., 2015). Internalization of negative stereotypes about ADHD may be particularly prevalent among boys and young men, who also report reluctance to seek help (Visser et al., 2024). However, a recent study (Jelinkova et al., 2024) reported much lower levels of self-stigma among youth with ADHD than previously published studies. These inconsistencies demonstrate the need for further investigation of the role of self-stigma among individuals with impulse control problems. Additionally, rather than focusing on disorder categories, investigating broader dimensions (i.e., the externalizing spectrum) may shed light on common underlying processes contributing to multiple forms of behavioral dysfunction and, in turn, ultimately inform transdiagnostic intervention strategies.

A shift toward understanding the role of self-stigma across broad psychopathology spectra rather than distinct disorders may prove especially useful. The present DSM-based classification system of psychopathology uses a categorical model to organize psychological disorders, implemented under the assumption that disorders are discrete entities with distinct etiology, course, and symptoms. However, extensive research has shown that psychopathology is not categorical in nature. Instead, almost all forms of psychopathology reflect extreme positions on continuously distributed dimensions (Markon et al., 2011). The externalizing spectrum, for example, encompasses impulse control disorders such as attention-deficit hyperactivity disorder, substance use disorders, oppositional defiant disorder, conduct disorder, and antisocial personality disorder. These conditions show high levels of comorbidity (Vaidyanathan et al., 2011; Waschbusch, 2002) and are best understood as differential expressions of a general proneness to impulse control problems that is expressed phenotypically as various behaviors and traits such as impulsivity, antisocial behavior, and substance use (Krueger et al., 2001, 2005). Thus, rather than being completely distinct entities, the externalizing factor represents a behavioral expression of an underlying vulnerability that contributes to all disorders along the externalizing spectrum.

It is important to identify influences on externalizing proneness which may be amenable to intervention. Self-stigma is a particularly intriguing candidate, given that it likely reflects the dynamic interplay of dispositional characteristics (e.g., tendencies toward negative appraisals) and exposure to broader environmental and sociocultural influences that maintain stigma of mental illness. Self-stigma may serve as a transdiagnostic cognitive process that helps explain the association between severe symptomatology and treatment-seeking intentions for a variety of presenting problems, rather than being specific to only a subset of disorders or conditions.

Societal attitudes related to specific impulse control problems, such as substance use disorders, suggest the public continues to endorse stigmatizing attitudes toward individuals with high levels of impulsive behaviors (Corrigan et al., 2009b; Lang and Rosenberg, 2017). The public views these individuals with disgust and perceives them to be dangerous and less worthy (Yang et al., 2017). Although considerable efforts have been dedicated to reducing this stigma, these beliefs have remained stable over time (Pescosolido et al., 2010). Given that public stigmatizing views are resistant to change, individuals with impulse control disorders may be particularly susceptible to internalizing these longstanding and discrediting messages (i.e., self-stigma). Specifically, the higher the level of externalizing behaviors an individual displays the greater the likelihood they will encounter negative societal stereotypes about persons with externalizing disorders, which may in turn increase the likelihood they internalize negative stereotypes related to these patterns. Self-stigma may subsequently decrease their treatment seeking intention to avoid additional shame or judgment from family, friends, or mental health providers.

The objective of the current study is to examine the relation between mental illness self-stigma, externalizing proneness (i.e., the general predisposition that underlies impulse control disorders), and treatment seeking intention. This study also examines the extent to which self-stigma mediates the relation between externalizing and the intention to seek treatment.

We hypothesized the following specific findings:

*H1*: General externalizing proneness will be positively associated with the intention to seek treatment, due to heightened negative affectivity.

*H2*: Externalizing proneness will be positively associated with mental illness self-stigma.

*H3*: Mental illness self-stigma will be negatively associated with the intention to seek treatment.

*H4a*: Mental illness self-stigma will partially mediate the relation between externalizing proneness and the intention to seek treatment.

*H4b*: This mediation will be strongest at the end-stage progression of self-stigma (i.e., as assessed by the “Harm to Self” subscale).

## Materials and methods

### Participants and procedure

Participants (*N* = 426) were recruited from an online, community sourcing platform (i.e., Prolific) in the United States (M_age_ = 34.10 years, range = 19–67, SD = 9.7 years). Inclusion criteria were selected from available pre-set Prolific options to identify individuals with a higher likelihood of externalizing problems in addition to broader indications of psychopathology. Specifically, participants were permitted to participate in the study if they endorsed all of the following, (1) use of alcohol, (2) ongoing mental health/illness/condition, (3) diagnosis of ADD/ADHD, and (4) smoking: tobacco or e-cigarettes. No specific exclusion criteria were applied. The use of Prolific in addition to this inclusion criteria increases the study’s internal and external validity, as participants reflect a nationally representative sample endorsing a range of externalizing presentations. Prolific also demonstrates higher quality data (e.g., reduced failed attention checks or ability to retain study information) compared to other online data collection platforms (Douglas et al., 2023). Thirty-two participants were excluded from analyses due to failing more than two attention checks (e.g., item repeats or “select answer choice ‘D’”); one attention check was included per measure.

Removal of these participants yielded a final analysis sample of 167 male, 223 female, and four participants who preferred not to specify their sex (*N* = 394). The vast majority (82%) identified as Caucasian, with the remainder as Black/African American (5.8%), Latin (5.1%), Asian American (4.6%), Other (1%), Middle Eastern/North African (0.8%), or Indigenous (0.5%). Approximately 30% of participants reported an income of $40,000 - $70,000 per year, followed by more than $100,000 per year (22.1%), $70,001 to $100,000 (19.8%), $20,000 to $40,000 (15.7%), less than $20,000 per year (8.4%), and Unknown (3.6%).

All participants provided informed consent, and procedures were approved by the university institutional review board (IRB). After participants consented to the study, they were directed to a secure Qualtrics survey. Measures were randomized to reduce the impact of participant fatigue or dropout. The average time to complete all instruments was approximately 25 min. Consistent with Proflic’s payment principles, participants were offered $12.00 per hour upon completion of the study (Prolific, 2023), and there were no penalties for those who declined to participate.

### Measures

#### Self-stigma of mental illness scale (SSMIS)

Self-stigma was assessed using the 20 item Self-Stigma of Mental Illness Scale-Short Form (SSMIS-SF; Corrigan et al., 2012). This measure includes four subscales each containing five items: Awareness, Acceptance, Application, and Harm to Self. Participants rate items on a scale from 0 (*strongly disagree*) to 9 (*strongly agree*). Previous studies have demonstrated convergent validity with other variables related to self-stigma (Corrigan et al., 2012; Schomerus et al., 2011; Rüsch et al., 2006). The SSMIS-SF showed good to excellent internal consistency in this sample (Awareness: *ω* = 0.97; Agreement: *ω* = 0.94; Application: *ω* = 0.85; Harm to Self: *ω* = 0.87).

#### Externalizing spectrum inventory (ESI)

The Externalizing Spectrum Inventory-Brief Form (ESI-BF; Patrick et al., 2013) is a 100-item self-report measure. The ESI-BF was developed to streamline the original 415-item ESI (ESI; Krueger et al., 2007) and is designed to measure a broad range of externalizing behaviors such as theft, fraud, physical and relational aggression, and drug use. Participants rate items on a four-point Likert scale from 0 (*false*) to 3 (*true*), with higher scores indicating higher externalizing behaviors. The measure yields a total score, along with scores on three higher-order dimensions (general externalizing, callous aggression, and substance abuse) and 23 lower-order scales. The ESI shows reliable scores across various samples including offender, community, and substance using adults (*α* above 0.85 for total and subscale scores) (Delfin et al., 2021; Patrick et al., 2013; Soe-Agnie et al., 2016). The current study focused on the general externalizing score, which demonstrated excellent reliability (*ω* = 0.95).

#### Mental help seeking intention scale (MHSIS)

The MHSIS contains three items designed to assess the intention to seek mental health services if an individual has a mental health concern. Participants rate items from 1 (*extremely unlikely*) to 7 (*extremely likely*). Scores are averaged for a maximum total score of 7 with higher scores indicating a higher likelihood to seek help. The MHSIS shows evidence of predictive validity of the actual intention to seek treatment (Hammer and Spiker, 2018). This measure demonstrated excellent internal consistency reliability in the current sample (*ω* = 0.97).

### Covariates

Self-esteem, self-efficacy, and internalizing symptoms were investigated as covariates, as it is plausible that the association between externalizing proneness and treatment-seeking intention is attributable to comorbid internalizing distress or the harmful consequences of self-stigma (diminished self-esteem and self-efficacy) rather than the direct impact of self-stigma.

#### New general self-efficacy scale (NGSE)

Self-efficacy was measured through the 8-item General Self-Efficacy Scale (NGSE; Chen et al., 2001). Participants rate items on a 5-point Likert-type scale from 1 (*strongly disagree*) to 5 (*strongly agree*), with higher scores indicating higher self-efficacy. This sample showed excellent internal consistency reliability (*ω* = 0.94).

#### Rosenberg self-esteem scale (RSES)

The Rosenberg Self-Esteem Scale (RSES; Rosenberg, 1965) is a 10-item self-report measure of self-esteem. The items assess self-competence and self-liking which index both positive and negative feelings about the self. Items are answered on a 4-point Likert-type scale from 1 (*Strongly Disagree*) to 4 (*Strongly Agree*), with higher scores indicating higher self-esteem. The current study used the unidimensional RSES total scores, which demonstrated excellent internal consistency (*ω* = 0.93).

#### Inventory of depression and anxiety symptoms (IDAS)

The Inventory of Depression and Anxiety Symptoms (IDAS; Watson et al., 2007) is a widely used self-report measure which yields dimensional scores on a range of internalizing and mood symptoms. The IDAS includes 99 items with 19 sub facets such as general depression, panic, and mania. Previous studies have shown evidence of good convergent and discriminant validity (Stasik-O'Brien et al., 2019; Watson et al., 2008). The present study focused on the General Depression subscale, which showed excellent internal consistency reliability in this sample (*ω* = 0.93).

## Data analytic approach

### Descriptive statistics and correlation analyses

Score distributions for all study variables were evaluated for skewness and kurtosis. Most variables displayed slight deviations from normality (skew = −0.08–0.1.68; kurtosis = −0.08–3.66). However, due to a high degree of positive skew for SSMIS scores, this measure was log-transformed. To address potential problems related to multicollinearity, continuous variables were mean centered. We performed bivariate correlational analyses to examine pairwise associations between externalizing proneness, the intention to seek treatment, and self-stigma. Additional analyses (correlations, t-tests, and ANOVAs) were performed to identify covariates, such as gender, self-esteem, self-efficacy, and depression, as well as the degree to which mean levels of study variables differed as a function of sociodemographic variables.

### Mediation analyses

After establishing support for the conceptual framework on the basis of significant zero-order associations between externalizing, treatment seeking, and self-stigma, mediational analyses were examined by including all four stages as simultaneous mediators in a structural equation model (SEM) mediational framework. SEM is a hypothesis driven method used to draw causal relations between a set of variables (Gunzler et al., 2013), but can also be applied to cross-sectional data to describe associations among variables without inferring directional effects. To carry out this SEM, mediational analyses were performed using the “lavaan” package in R (v. 0.6–7; Rosseel, 2012) using full information maximum likelihood (FIML) to account for missing data. The lavaan package was chosen to address potential issues with non-normal data (e.g., negative skew) and reduces the risk for Type I errors which may occur from running multiple, simple regressions for each self-stigma stage. Using a path analysis within an SEM allowed for the exploration of both direct and indirect relations between observed indicators in the proposed model. The indirect or mediating effect of the self-stigma stages was examined using 5,000 bootstrapped samples, creating 95% confidence intervals. A significant indirect effect was signified by a confidence interval that does not contain zero. It is important to note that although mediation analysis infers a causal relationship, the current cross-sectional research design cannot determine cause-and-effect relationships.

## Results

Descriptive statistics, internal consistencies (ω), and correlations between study variables are presented within Table 1. Contrary to Hypothesis 1, externalizing proneness was inversely correlated with the intention to seek treatment *r* = −0.10, *p* = 0.04. As predicted by Hypothesis 2, externalizing proneness demonstrated somewhat stronger positive relations with SSMIS Application *r* = 0.24, *p* < 0.001, and Harm to Self *r* = 0.23, *p* < 0.001, but negligible associations at the awareness or agreement stages. Contrary to the initial hypothesis, the magnitude of the correlation between externalizing and the most advanced stage of self-stigma (harm to self) did not differ from the association with the application stage, Steiger’s *Z* = 0.43, *p* = 0.66. Consistent with Hypothesis 3, self-stigma stage scores were negatively associated with the intention to seek treatment, with the exception of awareness, *rs* = −0.20, −0.17, −0.18, and −0.02, for SSMIS Application, Harm to Self, Agreement, and Awareness, respectively.

**Table 1:** Mean, SDs, internal consistency, and bivariate correlations of externalizing proneness, treatment-seeking intention, self-stigma (SSMIS) and covariates.

Variable	M	*SD*	ω	1	2	3	4	5	6	7	8	9
1. Externalizing Spectrum	206.76	45.11	0.95	--								
2. MHSIS	15.70	5.10	0.97	−0.10^*^	--							
3. Awareness (SSMIS)	44.92	22.85	0.97	−0.10^*^	−0.02	--						
4. Agreement (SSMIS)	22.73	13.26	0.94	0.04	−0.18^***^	−0.31^***^	--					
5. Application (SSMIS)	21.13	11.26	0.85	0.24^***^	−0.20^***^	−0.16^**^	0.51^***^	--				
6. Harm to Self (SSMIS)	21.67	12.98	0.87	0.23^***^	−0.17^***^	−0.16^**^	0.38^***^	0.80^***^	--			
7. Self esteem	25.52	6.90	0.93	−0.14^**^	0.21^***^	0.13^**^	−0.07	−0.43^***^	−0.52^***^	--		
8. Self-efficacy	28.54	7.02	0.94	−0.06	0.22^***^	0.05	−0.07	−0.41^***^	−0.46^**^	0.68^**^	--	
9. Depression	51.50	14.80	0.93	0.22^***^	−0.13^*^	−0.17^**^	0.09	0.36^**^	0.46^**^	−0.62^***^	−0.46^***^	--
10. Age	34.10	9.7	--	−0.01	0.09	−0.02	0.13^*^	−0.06	−0.09	0.06	0.01	−0.02

M, mean; SD, standard deviation; *ω*, omega (internal reliability statistic); Externalizing Spectrum, 100 item Externalizing Spectrum Inventory; MHSIS, 3 item Mental Help Seeking Intention Scale; SSMIS = Self-Stigma of Mental Illness Scale.

* *p* < 0.05.

** *p* < 0.01.

*** *p* < 0.001.

With regard to potential covariates, significant zero-order correlations were observed between study variables and self-efficacy, self-esteem, and depression (see Table 1). With respect to sociodemographic variables, externalizing was not associated with age or gender, nor were there significant differences in externalizing proneness as a function of race or income. No racial differences in levels of self-stigma were observed. However, women endorsed higher levels of the harm to self-stigma compared to men (*t* = 2.04, *p* = 0.04; Cohen’s d = 0.21). Levels of stigma differed across income groups for SSMIS Awareness, Application, and Harm to Self, Fs = 2.39, 3.28, and 3.60, *ps <*0.05. Treatment-seeking intention was not associated with age, gender, race, or income.

### Tests of mediation

After establishing support for the conceptual framework as indicated by significant zero-order associations between study variables, all four stages of self-stigma were included as concurrent mediators within an SEM framework. A saturated model was fit to the data.

**Figure 1:**
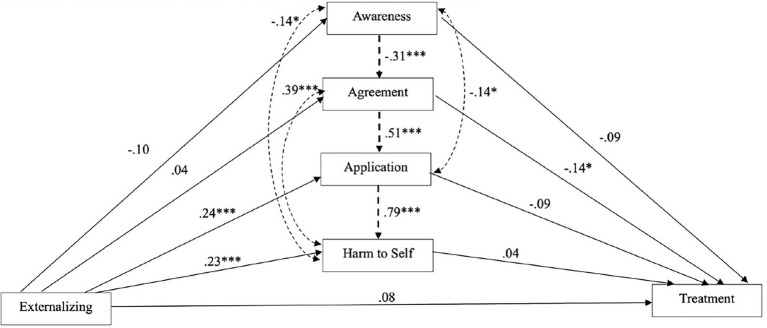
Structural equation mulitple mediation model with externalizing proneness predicting treatment-seeking intention via self-stigma (SSMIS) stages. Mediation analysis predicting the direct and indirect relations between externalizing proneness, stages of self-stigma, and treatment-seeking intention. Solid lines represent direct pathways; dotted lines represent covariances. Awareness = SSMIS Awareness scores; Agreement = SSMIS Agreement scores; Application = SSMIS Application scores; Harm to Self = SSMIS Harm to Self-scores; Treatment = MHSIS scores. * *p* < 0.05. ** *p* < 0.01. *** *p* < 0.001.

Results from the lavaan mediational analyses are represented visually in Figure 1. After including all variables in the model, the SEM model revealed that general externalizing proneness was no longer significantly associated with the intention to seek treatment (*b* = −0.29; *β* = −0.08; 95% CI [−0.19–0.04], SE = 0.06, *p* = 0.18). Contrary to Hypothesis 2, externalizing proneness was only associated with the later stages of self-stigma. Specifically, controlling for the covariance between the self-stigma stages, higher endorsement of externalizing proneness was significantly associated with higher scores on SSMIS Application (*b* = 0.12; *β* = 0.24; 95% CI [0.14–0.34], SE = 0.05; *p* < 0.001) and Harm to Self (*b* = 0.12; *β* = 0.23; 95% CI [0.12–0.33], SE = 0.05; *p* < 0.001), but was not related to awareness of self-stigma stereotypes (*b* = −0.07; *β* = −0.10; 95% CI [−0.22–0.02], SE = 0.06; *p* = 0.10) or self-stigma agreement (*b* = 0.02; *β* = 0.04; 95% CI [−0.07–0.14], SE = 0.05; *p* = 0.50).

With regard to the association between self-stigma and treatment seeking intention, higher levels of SSMIS Agreement were inversely associated with the intention to seek treatment (*b* = −0.45; *β* = −0.14; 95% CI [−0.28 – -0.001], SE = 0.07; *p* = 0.05), whereas the other stages of self-stigma were not related to treatment seeking, contrary to Hypothesis 3: SSMIS Awareness (*b* = −0.45; *β* = −0.09; 95% CI [−0.21–0.04], SE = 0.06; *p* = 0.19), Application (*b* = −0.70; *β* = −0.09; 95% CI [−0.28–0.10], SE = 0.10; *p* = 0.36), Harm to Self (*b* = −0.28; *β* = −0.04; 95% CI [−0.21–0.13], SE = 0.09; *p* = 0.65). Critically, none of the indirect pathways between externalizing proneness liability and treatment-seeking intention were significant, suggesting mediation did not occur as a function of self-stigma through the awareness (*b* = 0.33; *β* = 0.01; 95% CI [−0.01 – -0.03], SE = 0.01; *p* = 0.33), agreement (*b* = −0.02; *β* = −0.01; 95% CI [−0.02–0.01], SE = 0.01; *p* = 0.52), application (*b* = −0.08; *β* = −0.02; 95% CI [−0.07–0.03], SE = 0.02; *p* = 0.38), or harm to self (*b* = −0.03; *β* = −0.01; 95% CI [−0.05–0.03], SE = 0.02; *p* = 0.66) stages of self-stigma, indicating neither Hypothesis 4a nor Hypothesis 4b was supported. Nonetheless, the total effect was marginally significant (*b* = −0.39; *β* = −0.10; 95% CI [−0.21–0.01], SE = 0.20, *p* = 0.06), which suggests the indirect and direct pathways accounted for variance in treatment-seeking intention to a small degree. ^1^

## Discussion

The current study examined the association between mental illness self-stigma, externalizing proneness, and the intention to seek treatment. At the zero-order level, externalizing proneness was weakly correlated with lower treatment-seeking intention and greater application and harm to self of self-stigma. However, when all four stages were examined as concurrent mediators, these associations were weaker and largely nonsignificant, indicating that the presence of self-stigma did not account for the lower treatment-seeking intention among individuals with impulse control problems.

Despite the nonsignificant findings for multiple mediation, the current study yielded important results that warrant mentioning. Specifically, externalizing proneness was associated with the application and harm to self-stages of self-stigma scores, which suggests individuals with higher levels of externalizing problems are more likely to internalize negative stereotypes of mental illness when compared to individuals with lower levels of externalizing problems. These findings were consistent with hypotheses and parallel previous literature (Ali et al., 2017), which found high levels of self-stigma among specific externalizing disorders. Thus, the results of the current study build on the empirical literature by demonstrating that self-stigma is ubiquitous across the spectrum of impulse control problems rather than being specific to only certain disorders, broadening our understanding of how self-stigma functions across a key spectrum of psychopathology.

Unexpectedly, the application stage instead of the harm to self-stage demonstrated the strongest association with externalizing proneness. Likely, the deleterious effects of self-stigma emerge once an individual applies harmful stereotypes about mental illness to themselves, even before hopelessness or harm to self occurs, at least for individuals with externalizing forms of psychopathology. Regarding mediation, although the awareness (*β* = −0.09), application (*β* = −0.09), and harm to self (*β* = −0.04) stages were not significantly related to treatment-seeking intention within the SEM model, a significant, inverse relation was found between agreement with negative stereotypes and the intention to seek treatment (*β* = −0.14). These results are consistent with self-stigma literature, which suggests individuals who agree with certain mental illness stereotypes (i.e., individuals with mental illness are unpredictable) concurrently deny the willingness to seek treatment (Mojtabai, 2010). In other words, individuals may forgo treatment to avoid the label associated with mental health concerns (i.e., They are crazy, dangerous, or dirty; Corrigan, 2004).

Critically, however, the lack of significant mediation in the present study suggests self-stigma largely does not explain lower treatment seeking intention among individuals high in externalizing proneness. Treatment seeking behaviors are determined by a complex set of societal and ecological factors, and individual-level stigma may not be a particularly strong predictor of such behaviors for individuals with impulse control problems. For example, a range of social determinants are associated with mental health treatment underutilization, including geographical isolation, lack of transportation or childcare, and inadequate insurance coverage or other financial concerns (Tambling et al., 2022). Likewise, externalizing problems are also associated with sociodemographic disadvantage (Derzon, 2010), so it plausible that factors like poverty better explain the association between impulse control problems and reduced treatment seeking.

Nevertheless, it is likely that self-stigma is associated with other deleterious outcomes for individuals with externalizing problems, which should be explored in future research. Self-stigma may serve as a stronger mediator for outcomes with a strong individual difference component as compared to something like treatment-seeking, which has many external, structural-level influences. For example, it should be investigated whether self-stigma is associated with worsening mental health symptoms, poorer medication adherence, or reduced treatment effectiveness for individuals with impulse control problems, as has been reported for patients with internalizing and psychotic disorders (Ritsher and Phelan, 2004; Uhlmann et al., 2014). Given that self-stigma appears to be a transdiagnostic and modifiable target for therapeutic intervention, it is plausible that providers can address self-stigma within common therapeutic interventions if these individuals demonstrate a willingness to seek treatment. Suppose we conceptualize self-stigma as a cognitive style (i.e., a mode of thinking or evaluating information) in conjunction with broader societal influences, cognitive restructuring may prove helpful as a strategy for reducing internalized stigma and its associated mental health impacts. Extensive literature shows that treatment modalities such as cognitive behavioral therapy (CBT) or acceptance and commitment therapy (ACT) effectively reduce unhelpful thinking patterns (Goldin et al., 2016; Hofmann and Smits, 2008), so clinicians may be able to integrate discussions of self-stigma and challenging such cognitions into existing ESTs alongside work aimed at developing new programs specifically for self-stigma (e.g., Corrigan and Shapiro, 2010; Mittal et al., 2012).

### Limitations and future directions

A number of limitations to the current study warrant mentioning. It must be acknowledged that observed effect sizes for the current study were small; however, any significant finding should be considered salient and an important target to increase treatment use, as a delay or reluctance toward mental health treatment exacerbates health disparities and leads to significant impairment at the level of the individual. Although the current study implemented meditational analyses, it cannot make causal claims as the study design is cross-sectional. A longitudinal study design may be more appropriate to evaluate self-stigma and its impact on treatment-seeking. The reliance on self-report measures is another limitation, and future research should seek to take a multi-method approach. In particular, the treatment-seeking measures used may not mirror the reality of an individual’s actual decision to seek treatment. Future research may benefit from assessing treatment use behaviors rather than intention using objective measures (e.g., health records or therapy session frequency). Finally, the use of Prolific may have limited our ability to identify a sample with severe externalizing psychopathology. For example, our sample was largely economically advantaged (only 24.1% of the sample earned less than $40,000 per year) and thus may reflect a community sample with relatively low impairment. Future studies should examine these associations within clinical samples.

Notwithstanding these limitations, this study demonstrates that self-stigma exists across the spectrum of externalizing problems. These findings provide critical information to clinicians and researchers alike, as self-stigma is a transdiagnostic, modifiable target to decrease the deleterious effects of public stigma for individuals who struggle with externalizing problems. Future research should examine a range of relevant mental health outcomes, as well as the interaction between self-stigma and social determinants of health to develop more comprehensive models of stigma and impulse control problems.

## Data Availability

The datasets presented in this study can be found in online repositories. The names of the repository/repositories and accession number(s) can be found in the article/Supplementary material.

## Footnotes

1 When accounting for sex as a covariate in the model, results were unchanged and all tests of mediation remained non-significant.
