# Supplementary material for: The role of self-stigma in mediating the association between externalizing and treatment-seeking intention
Source: Front Psychol. 2025 Dec 12;16:1686583. doi: 10.3389/fpsyg.2025.1686583 (PMC12742856; doi:10.3389/fpsyg.2025.1686583)
Supplement: Supplementary file 1 [file Data_Sheet_1.docx]

The data that support the findings of this study are available at <https://osf.io/kauj8/?view_only=84176a24ed424a88bb9535dea7398ca9>
