# Supplementary material for: The role of self-stigma in mediating the association between externalizing and treatment-seeking intention
Source: Front Psychol. 2025 Dec 12;16:1686583. doi: 10.3389/fpsyg.2025.1686583 (PMC12742856; doi:10.3389/fpsyg.2025.1686583)
Supplement: Supplementary file 2 [file Table_1.DOCX]

**Table S1**

*Mean, SDs, and T-Test Results of Externalizing Spectrum, Treatment-Seeking Intention, Self-Stigma (SSMIS) by Sex*

| **Externalizing Spectrum** | **M** | **SD** | ***t*** | ***p*** | ***d*** |
| --- | --- | --- | --- | --- | --- |
| Female | 204.07 | 42.16 | -1.55 | .12 | -.16 |
| Male | 211.22 | 48.66 |  |  |  |
| **MHSIS** | **M** | **SD** | ***t*** | ***p*** | ***d*** |
| Female | 5.33 | 1.58 | 1.30 | .20 | .13 |
| Male | 5.10 | 1.85 |  |  |  |
| **Total Self-Stigma** (SSMIS) | **M** | **SD** | ***t*** | ***p*** | ***d*** |
| Female | 2.01 | .18 | 1.74 | .08 | .18 |
| Male | 1.98 | .20 |  |  |  |
| **Awareness** (SSMIS) | **M** | **SD** | ***t*** | ***p*** | ***d*** |
| Female | 1.56 | .33 | -1.57 | .12 | -.16 |
| Male | 1.61 | .31 |  |  |  |
| **Agreement** (SSMIS) | **M** | **SD** | ***t*** | ***p*** | ***d*** |
| Female | 1.30 | .22 | -.01 | .99 | .00 |
| Male | 1.30 | .24 |  |  |  |
| **Application** (SSMIS) | **M** | **SD** | ***t*** | ***p*** | ***d*** |
| Female | 1.28 | .21 | .83 | .41 | .09 |
| Male | 1.26 | .22 |  |  |  |
| **Harm to Self** (SSMIS) | **M** | **SD** | ***t*** | ***p*** | ***d*** |
| Female | 1.29 | .24 | 2.04 | .04 | .21 |
| Male | 1.24 | .24 |  |  |  |

*Note*. M= mean; SD = standard deviation; Externalizing Spectrum = Externalizing Spectrum Inventory; MHSIS = 3 item Mental Help Seeking Intention Scale; SSMIS = Self-Stigma of Mental Illness Scale.

**Table S2**

*Mean, SDs, and T-Test Results of Externalizing Spectrum, Treatment-Seeking Intention, Self-Stigma (SSMIS) by Race*

| **Externalizing Spectrum** | **M** | **SD** | ***F*** | ***p*** | ***η2*** |
| --- | --- | --- | --- | --- | --- |
| White | 206.80 | 44.98 | .20 | .40 | .01 |
| Black | 193.90 | 36.74 |  |  |  |
| Latin | 205.82 | 52.64 |  |  |  |
| **MHSIS** | **M** | **SD** | **F** | ***p*** | ***η2*** |
| White | 5.23 | 1.68 | .71 | .49 | .00 |
| Black | 5.60 | 1.86 |  |  |  |
| Latin | 5.02 | 1.96 |  |  |  |
| **Total Self- Stigma** (SSMIS) | **M** | **SD** | **F** | ***p*** | ***η2*** |
| White | 2.00 | .19 | .19 | .83 | .00 |
| Black | 1.99 | .20 |  |  |  |
| Latin | 2.02 | .18 |  |  |  |
| **Awareness** (SSMIS) | **M** | **SD** | **F** | ***p*** | ***η2*** |
| White | 1.59 | .32 | .07 | .94 | .00 |
| Black | 1.57 | .27 |  |  |  |
| Latin | 1.57 | .26 |  |  |  |
| **Agreement** (SSMIS) | **M** | **SD** | **F** | ***p*** | ***η2*** |
| White | 1.29 | .22 | .16 | .75 | .00 |
| Black | 1.27 | .27 |  |  |  |
| Latin | 1.32 | .19 |  |  |  |
| **Application** (SSMIS) | **M** | **SD** | **F** | ***p*** | ***η2*** |
| White | 1.27 | .21 | .16 | .85 | .00 |
| Black | 1.25 | .21 |  |  |  |
| Latin | 1.25 | 21 |  |  |  |
| **Harm to Self** (SSMIS) | **M** | **SD** | **F** | ***p*** | ***η2*** |
| White | 1.27 | .23 | .43 | .65 | .00 |
| Black | 1.22 | .23 |  |  |  |
| Latin | 1.28 | .27 |  |  |  |

*Note*. M= mean; SD = standard deviation; Externalizing Spectrum = 100 item Externalizing Spectrum Inventory; MHSIS = 3 item Mental Help Seeking Intention Scale; SSMIS = Self-Stigma of Mental Illness Scale.

**Table S3**

*Mean, SDs, and One Way ANOVA Results of Externalizing Spectrum, Treatment-Seeking Intention, Self-Stigma (SSMIS) by Income*

| **Externalizing Spectrum** | **M** | **SD** | **F** | ***p*** |  | ***η2*** |
| --- | --- | --- | --- | --- | --- | --- |
| less than $20,000 per year | 217.42 | 44.69 | 1.74 | .13 |  | .02 |
| $20,001 - $40,000 per year | 209.39 | 44.92 |  |  |  |  |
| $40,001 - $70,000 per year | 212.69 | 46.26 |  |  |  |  |
| $70,001 - $100,000 per year | 200.92 | 44.37 |  |  |  |  |
| more than $100,000 per year | 200.10 | 44.77 |  |  |  |  |
| **MHSIS** | **M** | **SD** | **F** | ***p*** |  | ***η2*** |
| less than $20,000 per year | 4.91 | 1.91 | 1.56 | .17 |  | .02 |
| $20,001 - $40000 per year | 4.81 | 1.87 |  |  |  |  |
| $40,0001 - $70000 per year | 5.26 | 1.73 |  |  |  |  |
| $70,000 - $100,000 per year | 5.43 | 1.51 |  |  |  |  |
| **Overall Stigma (SSMIS)** | **M** | **SD** | **F** | ***p*** |  | ***η2*** |
| less than $20000 per year | 2.07 | 2.07 | 4.03 | .04 |  | .05 |
| $20,001 - $40000 per year | 2.04 | .19 |  |  |  |  |
| $40,001 - $70000 per year | 2.03 | .19 |  |  |  |  |
| $70,001 - $100000 per year | 1.95 | .21 |  |  |  |  |
| more than $100,000 per year | 1.96 | .18 |  |  |  |  |
| **Awareness (SSMIS)** | **M** | **SD** | **F** | ***p*** |  | ***η2*** |
| less than $20,000 per year | 1.58 | .20 | 2.39 | .04 |  | .02 |
| $20,001 - $40,000 per year | 1.54 | .33 |  |  |  |  |
| $40,001 - $70,000 per year | 1.52 | .39 |  |  |  |  |
| $70,001 - $100,000 per year | 1.63 | .33 |  |  |  |  |
| more than $100,000 per year | 1.65 | .24 |  |  |  |  |
| **Agreement (SSMIS)** | **M** | **SD** | **F** | ***p*** |  | ***η2*** |
| less than $20,000 per year | 1.33 | .19 | 1.72 | .13 |  | .02 |
| $20,001 - $40,000 per year | 1.33 | .25 |  |  |  |  |
| $40,001 - $70,000 per year | 1.32 | .24 |  |  |  |  |
| $70,001 - $100,000 per year | 1.25 | .21 |  |  |  |  |
| more than $100,000 per year | 1.27 | .22 |  |  |  |  |
| **Application (SSMIS)** | **M** | **SD** | **F** | ***p*** |  | ***η2*** |
| less than $20,000 per year | 1.39 | .20 | 3.28 | .01 |  | .04 |
| $20,001 - $40,000 per year | 1.28 | .22 |  |  |  |  |
| $40,001 - $70,000 per year | 1.27 | .22 |  |  |  |  |
| $70,001 - $100,000 per year | 1.23 | .21 |  |  |  |  |
| more than $100,000 per year | 1.24 | .21 |  |  |  |  |
| **Harm to Self(SSMIS)** | **M** | **SD** | **F** | ***p*** |  | ***η2*** |
| less than $20,000 per year | 1.35 | .23 | 3.60 | .003 |  | .04 |
| $20,001 - $40,000 per year | 1.30 | .24 |  |  |  |  |
| $40,001 - $70,000 per year | 1.29 | .25 |  |  |  |  |
| $70,001 - $100,000 per year | 1.22 | .23 |  |  |  |  |
| more than $100,000 per year | 1.21 | .22 |  |  |  |  |

*Note*. M= mean; SD = standard deviation; Externalizing Spectrum = Externalizing Spectrum Inventory; MHSIS = 3 item Mental Help Seeking Intention Scale; SSMIS = Self-Stigma of Mental Illness Scale.
